# Clinical characteristics and prognosis of patients with multiple intracranial aneurysms living on the Tibetan Plateau of China

**DOI:** 10.1177/03000605241306870

**Published:** 2024-12-29

**Authors:** Dongliang Wang, Jiuqi Wuli, Xudong Cao, Bin Wang, Zeng Ren, Yu Weng, Kexue Wu

**Affiliations:** 1 Department of Neurosurgery, Peking University People’s Hospital, Beijing, China; 2 Department of Neurosurgery, Tibet Autonomous Region People’s Hospital, Lhasa, China

**Keywords:** Intracranial aneurysm, Tibetan Plateau, multiple intracranial aneurysms, single intracranial aneurysm, hemorrhage, internal carotid artery

## Abstract

**Objectives:**

Although there is no consensus on the difference in rupture rates between multiple intracranial aneurysms (MIAs) and single intracranial aneurysms (SIAs), patients with MIAs may have poorer outcomes after rupture than patients with SIAs. In this study, we aimed to analyze differences in clinical characteristics between MIAs and SIAs and to evaluate the prognosis of patients with MIAs on the Tibetan Plateau who received different clinical treatments.

**Methods:**

We retrospectively analyzed the clinical data of 68 patients with MIAs and 68 patients with SIAs admitted to Tibet Autonomous Region People’s Hospital. Univariate and multivariate analyses were used for data analysis.

**Results:**

Compared with patients who had SIAs, those with MIAs were more likely to be female, smokers, alcohol drinkers, and hypertensive. The difference between the two groups in terms of size of the ruptured aneurysms was statistically significant. No significant differences in treatment effects were observed between patients with SIAs and those with MIAs at 3, 6, and 12 months.

**Conclusions:**

Among patients living on the Tibetan Plateau, those with MIAs were more likely to be female, smokers, alcohol drinkers, and hypertensive. Endovascular treatment was superior to conservative management but not significantly better than craniotomy.

## Introduction

Intracranial aneurysms are abnormal dilations of the intracranial vessels and are the leading cause of hemorrhagic stroke, accounting for 85% of subarachnoid hemorrhages (SAHs).^1^ The incidence of intracranial aneurysms is approximately 3.2% in adults.^2^ The etiology of intracranial aneurysms remains unclear. Currently, most scholars believe that their occurrence is owing to a combination of multiple factors, including acquired changes in hemodynamics, vascular structure, and congenital genetic problems.^3^

Multiple intracranial aneurysms (MIAs) involve two or more intracranial vessels. According to epidemiological research, the incidence of MIAs among all intracranial aneurysms is 7% to 35%.^4,5^ Several studies have reported that the rate of intracranial aneurysm rupture in patients with MIAs is significantly higher than that in patients with single intracranial aneurysms (SIAs).^6,7^ However, research on this topic has yielded inconclusive results, with some studies suggesting that the difference in rupture rates may be reversed or that there is no significant difference.^8^ It is more challenging to diagnose and treat MIAs than SIAs. Previous evidence has also indicated that patients with MIAs may have poorer outcomes after rupture than patients with SIAs.^9,10^ Moreover, the surgical treatment strategies and prognoses for patients with MIAs differ significantly from those for patients with SIAs. Therefore, exploring the differences in epidemiological characteristics between patients with MIAs and SIAs is necessary, and determining whether an interaction exists between morbidity type and treatment modality is important to improve treatment effectiveness.

The Tibetan Plateau is one of the three polar regions on Earth (the others being the Arctic and Antarctic). The plateau has an average altitude of over 4000 m, low oxygen and air pressure, intense radiation, and a cold and dry climate.^11^ For people living on the plateau, blood flow in the brain increases and cerebral blood flow accelerates to compensate for the low-oxygen environment.^12^ The diet of most Tibetans is high in salt, fat, and cholesterol and low in vitamins.^13^ Therefore, Tibetans are more prone to high blood viscosity, fragility, and hardening of the blood vessel walls, which may be associated with cerebral aneurysm formation.

In previous studies, age, female sex, arterial hypertension, tobacco use, and other clinical indicators used in routine blood and biochemical tests have been consistently reported to be associated with MIAs.^8,14–16^ Research on MIAs in the Tibetan population is scarce, and whether specific risk factors for MIA exist in this region needs to be investigated. Endovascular treatment of aneurysms is an accepted standard treatment.^17,18^ Other treatment methods, such as conservative treatment and craniotomy, are still used in clinical practice. The clinical endovascular treatment approach for patients with MIAs living on the Tibetan Plateau is yet to be extensively discussed in the neurosurgical literature.

In this study, we aimed to analyze patients living on the Tibetan Plateau with intracranial aneurysms and the differences in clinical characteristics between patients with MIAs and SIAs in Tibet. Furthermore, we examined whether morbidity type (MIAs vs. SIAs) and treatment modality (conservative, craniotomy, or endovascular treatment) influenced the effectiveness of endovascular treatment based on the modified Rankin scale (MRS) score. Additionally, we investigated the effects of these treatment modalities on MIA prognosis.

## Methods

### Ethical approval

The Tibet Autonomous Region People’s Hospital Ethics Committee approved the study (approval no. ME-TBHP-22-30) on 26 July 2022. All participants or their families provided written informed consent for possible future data use in research before inclusion. We de-identified all patient details. All procedures were performed in accordance with the principles of the Helsinki Declaration of 1975 as revised in 2013.

### Study population

**Figure 1.**
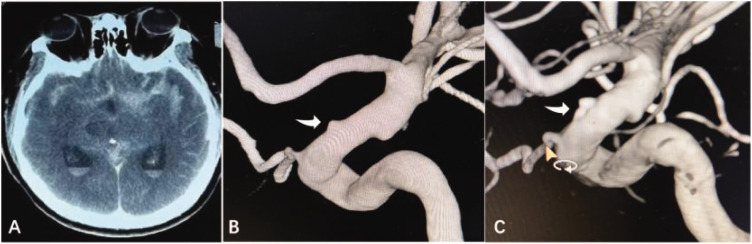
Computed tomography (CT) and digital subtraction angiography (DSA) imaging of a representative case. (a) CT showing subarachnoid hemorrhage (SAH). (b) DSA showing a small blood blister-like aneurysm (BBA; white arrow) of the supraclinoid internal carotid artery (ICA) and (c) BBA was treated using stent-assisted coil embolization (white arrow).

We retrospectively analyzed the medical records of patients with MIA admitted to the Department of Neurosurgery, Tibet Autonomous Region People’s Hospital, between June 2019 and May 2022. For controls, 68 patients with SIAs were randomly selected concurrently with patients who had MIAs. We included patients living in Tibet for an extended period (at least 10 years); patients with cerebral aneurysm confirmed based on computed tomography angiography (CTA)/magnetic resonance angiography (MRA)/digital subtraction angiography (DSA) examinations (Figure 1(a) and 1(b)); patients between 18 and 80 years of age; those with complete clinical data, and patients who agreed to use their hospitalization data for research and signed an informed consent form. The exclusion criteria were as follows: (1) SAH but no aneurysm identified on CTA or DSA; (2) presence of arteriovenous malformation, Moyamoya disease, or brain abscess; and (3) incomplete data and lack of cooperation with researchers.

### Data collection

**Figure 2.**
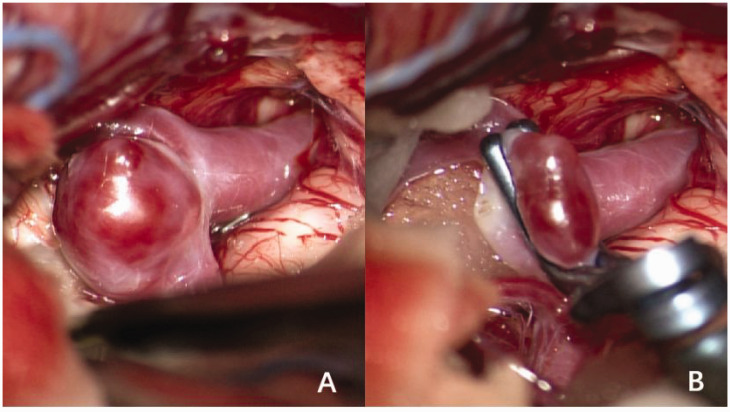
Craniotomy clipping of the cerebral aneurysm. (a) Middle cerebral artery (MCA) bifurcation aneurysm and (b) clipping of the MCA bifurcation aneurysm.

We collected the demographic characteristics and clinical information of all participants. Patients’ demographic characteristics included age, sex, race and ethnicity, relevant past medical history (hypertension, hyperlipidemia, diabetes mellitus, and hyperhemoglobinemia), and smoking and drinking habits. We collected clinical information, including the Fisher grade, Hunt and Hess scale score, aneurysm features (location, size, number, and morphology of the ruptured aneurysm), treatment (conservative, craniotomy, Figure 2(a) and 2(b); endovascular, Figure 1(c)), and MRS scores.

### Diagnostic methods and treatment protocols

All enrolled patients underwent cranial CTA and DSA examinations. Patients with unruptured aneurysms are generally screened according to high-risk groups or physical examinations. For patients whose first DSA examination was negative, three-dimensional angiography was performed 2 weeks later. The diagnosis of a blood blister-like aneurysm (BBA) at our center was based on the following criteria: 1) in the dorsal, non-branching site of the internal carotid artery (ICA), 2) small aneurysms (primary size, usually <3 mm) lacking an identifiable neck, and 3) thin-walled, with subsequent DSA showing rapid changes in the aneurysm.^19^

Based on the patient’s situation and local customs, the following treatment protocols were generally implemented. (1) For unruptured aneurysms, the patient’s family was informed about the risk of rupture and surgery. For patients managed conservatively, follow-up with CTA or MRA at regular intervals was recommended. Treatment decisions were mainly based on the wishes of the family. (2) For multiple aneurysms (Supplemental Figure 1), the ruptured aneurysm was treated first, and other aneurysms were treated simultaneously or followed up. (3) Endovascular treatment was administered for all BBAs. (4) When accompanied by an intracerebral hematoma, craniotomy was performed. (5) For patients who could undergo either craniotomy or endovascular treatment, the choice of treatment was based on the patient’s condition and decided by at least two senior neurosurgeons.

### Statistical analysis

All statistical analyses were performed using IBM SPSS version 26.0 (IBM Corp., Armonk, NY, USA). Continuous data are reported as mean ± standard deviation, and categorical variables are expressed as number and percentage. The independent samples *t*-test and chi-square test were used for continuous and categorical variables, respectively. Multivariate linear regression analyses were performed to identify the predictors of prognostic outcomes among patients with MIAs. The interaction between treatment and patients (SIAs or MIAs) was examined using two-way analysis of variance (ANOVA). Statistical significance was set at p < 0.05. The reporting of this study conforms to the Strengthening the Reporting of Observational Studies in Epidemiology (STROBE) guidelines.^20^

## Results

### Demographic and clinical characteristics of participants

This study included 136 Chinese patients with SIAs (n = 68) or MIAs (n = 68; 157 aneurysms in total) living on the Tibetan Plateau. The mean age of all participants was 51.0 ± 10.4 years. Significant differences were observed in the percentage of female patients (MIAs, 75%; SIAs, 58.8%; p < 0.05). A significantly higher prevalence of MIAs than of SIAs was observed among patients with a history of smoking (64.7% vs. 35.3%; p = 0.001), alcohol consumption (73.5% vs. 22.1%; p < 0.001), and hypertension (64.7% vs. 35.3%; p < 0.001). The difference in the size of ruptured aneurysms between the two groups was statistically significant (6.4 ± 1.6 mm vs. 7.6 ± 1.8 mm; p < 0.001). Regarding age, history of diabetes, blood test values, Fisher grade, Hunt and Hess scale score, aneurysm location, treatment, or MRS score, no significant differences were found between patients with MIAs and SIAs.

MIAs accounted for 22% (68/309) of all aneurysms during the study period. Of patients with MIAs, 51 (37.5%) had two aneurysms, 14 (10.3%) had three aneurysms, and 3 (2.2%) had four or more aneurysms. All patients underwent one of the following three treatments: conservative treatment (32.4%, n = 44), endovascular treatment (48.5%, n = 66), and craniotomy (19.1%, n = 26). Table 1 shows the clinical features, ruptured aneurysm characteristics, and treatment information of patients with SIAs and MIAs. The details of all aneurysms in patients with MIAs are summarized in Supplemental Table 1. The mean aneurysm size was 6.3 ± 1.8 mm. Regarding the location of the aneurysms, 62 (39.5%) were located in the middle cerebral artery (MCA), 56 (35.7%) were in the ICA, 30 (19.1%) were in the anterior cerebral artery (ACA), and nine (5.7%) were in the vertebral artery (Figure 1).

**Table 1.** Clinical features, characteristics of single or largest aneurysm, and treatment of patients with SIA and MIA.

Variables	Total (n = 136)	Patients with SIA (n = 68)	Patients with MIA (n = 68)	p-value
Sex, n (%)				0.045*
Male	45 (33.1)	28 (41.2)	17 (25.0)	
Female	91 (66.9)	40 (58.8)	51 (75.0)	
Age (years), mean ± SD	51.0 ± 10.4	49.8 ± 9.7	52.2 ± 10.9	0.183
History of smoking, n (%)	68 (50.0)	24 (35.3)	44 (64.7)	0.001**
History of drinking, n (%)	65 (47.8)	15 (22.1)	50 (73.5)	<0.001***
Diabetes, n (%)	66 (48.5)	34 (50.0)	32 (47.1)	0.864
Hypertension, n (%)	68 (50.0)	24 (35.3)	44 (64.7)	0.001**
Blood test values				
HB (mg/dL), mean ± SD	153.5 ± 27.6	153.4 ± 28.4	153.7 ± 27.0	0.957
PCV (fL), mean ± SD	44.6 ± 7.7	45.4 ± 7.2	43.9 ± 8.1	0.252
MCHC (g/dL), mean ± SD	331.4 ± 40.8	327.5 ± 56	335.3 ± 14.5	0.266
PLT (n/L), mean ± SD	3.3 ± 35.4	0.2 ± 0.1	6.3 ± 49.8	0.325
PT (s), mean ± SD	13.3 ± 1.2	13.4 ± 1.1	13.3 ± 1.4	0.789
FIB (g/L), mean ± SD	3.3 ± 1.0	3.4 ± 1.0	3.2 ± 0.9	0.323
D-dimer (μg/L), mean ± SD	2.4 ± 5.8	2.7 ± 7.9	2.0 ± 2.3	0.499
Fisher grade, mean ± SD	1.4 ± 1.3	1.3 ± 1.2	1.4 ± 1.3	0.541
Hunt and Hess scale grade, mean ± SD	2.1 ± 0.8	2.2 ± 0.8	2.0 ± 0.7	0.180
Number of aneurysms, n (%)				
1	68 (50.0)	68 (100.0)	NA	
2	51 (37.5)	NA	51 (75.0)	
3	14 (10.3)	NA	14 (20.6)	
4 or more	3 (2.2)	NA	3 (4.4)	
Ruptured aneurysm, n (%)	115 (84.6)	59 (86.8)	56 (82.4)	0.261
Location, n (%)				0.139
ACA	23 (16.9)	11 (16.2)	12 (17.6)	
ICA	58 (85.3)	33 (48.5)	25(36.8)	
MCA	28 (41.2)	12 (17.6)	16 (23.5)	
VBA	6 (4.4)	3 (4.4)	3 (4.4)	
Size (mm), mean ± SD	7.0 ± 1.8	6.4 ± 1.6	7.6 ± 1.8	<0.001***
BBAs, n (%)	17 (12.5)	10 (14.7)	7 (10.3)	0.124
Aneurysm with hematoma, n (%)	14 (10.3)	6 (8.8)	8 (11.8)	0.778
Treatment method, n (%)				0.739
Conservative treatment	44 (32.4)	20 (29.4)	24 (35.3)	
Endovascular treatment	66 (48.5)	35 (51.4)	31 (45.6)	
Craniotomy	26 (19.1)	13 (19.1)	13 (19.1)	
MRS score, mean ± SD				
3 months	2.3 ± 1.6	2.4 ± 1.4	2.3 ± 1.8	0.523
6 months	2.0 ± 1.8	2.0 ± 1.7	2.0 ± 2.0	0.963
12 months	1.7 ± 2.0	1.7 ± 2.0	1.7 ± 2.1	0.900

SIA, single intracranial aneurysm; MIA, multiple intracranial aneurysms; HB, hemoglobin; PCV, packed cell volume; MCHC, mean corpuscular hemoglobin concentration; PLT, plateletcrit; PT, prothrombin time; FIB, fibrinogen; ACA, anterior cerebral artery; ICA, internal carotid artery; MCA, middle cerebral artery; VBA, vertebral artery; BBAs, blood blister-like aneurysms; MRS, modified Rankin scale; NA, not applicable; SD, standard deviation.

### Effects of morbidity type and treatment modality on the MRS score

The effects of morbidity type (MIAs vs. SIAs) and treatment modality (conservative, craniotomy, or endovascular) on prognosis based on the MRS score were examined using between-patient ANOVA (Table 2). The MRS results indicated that the treatment modality significantly affected prognosis after 1, 6, and 12 months of endovascular treatment (1 month: F = 8.313, p < 0.05; 6 months: F = 17.583, p < 0.05; 12 months: F = 22.100, p < 0.05). The most significant difference in outcomes was observed between conservative and endovascular treatment modalities; the worst recovery was associated with conservative treatment and the best recovery with endovascular treatment. The interaction effects of both treatment modalities (1 month: F = 2.260; 6 months: F = 0.799; 12 months: F = 0.305) and type of morbidity (MIAs *vs*. SIAs) on MRS scores were not significant (1 month: F = 0.095; 6 months: F = 0.008; 12 months: F = 0.007).

**Table 2.** Description of morbidity type and treatment modality based on Fisher grade and MRS score over time.

Time period	Morbidity type	Treatment modality	n	Mean ± SD
3-month MRS	SIA	Conservative	20	2.80 ± 1.64
		Endovascular treatment	35	2.20 ± 1.35
		Craniotomy	13	2.46 ± 1.44
	MIA	Conservative	24	3.13 ± 1.85
		Endovascular treatment	31	1.39 ± 1.43
		Craniotomy	13	2.69 ± 1.38
6-month MRS	SIA	Conservative	20	2.95 ± 2.24
		Endovascular treatment	35	1.43 ± 1.22
		Craniotomy	13	2.08 ± 1.19
	MIA	Conservative	24	3.21 ± 2.13
		Endovascular treatment	31	0.94 ± 1.36
		Craniotomy	13	2.23 ± 1.59
12-month MRS	SIA	Conservative	20	2.95 ± 2.50
		Endovascular treatment	35	0.89 ± 1.31
		Craniotomy	13	1.69 ± 1.80
	MIA	Conservative	24	3.13 ± 2.31
		Endovascular treatment	31	0.55 ± 1.21
		Craniotomy	13	1.77 ± 2.01

SIA, single intracranial aneurysm; MIA, multiple intracranial aneurysm; MRS, modified Rankin scale.

### Analysis of treatment modality effects on the MRS score in patients with MIAs

The effects of the three treatment modalities were compared in the 68 patients with MIAs at three time points (3, 6, and 12 months) (Table 3). The results showed that when conservative management was used as a reference, endovascular treatment showed significant differences in MRS scores at all three time points (all p < 0.001). However, no differences were found in patients who underwent craniotomy. These results were consistent with those of the unadjusted model after adjusting for age, sex, history of smoking, history of drinking, diabetes, and hypertension.

**Table 3.** Effects of treatment modality on MRS score based on multivariate regression.

Variable	Model	Treatment	Estimate	95% CI	*p*-value
3-month MRS	Unadjusted	Endovascular treatment	1.741	(0.874, 2.607)	<0.001***
		Craniotomy	0.862	(−0.236, 1.960)	0.122
	Adjusted	Endovascular treatment	1.816	(0.842, 2.790)	<0.001***
		Craniotomy	0.993	(−0.386, 2.372)	0.155
6-month MRS	Unadjusted	Endovascular treatment	1.964	(0.990, 2.937)	<0.001***
		Craniotomy	1.125	(−0.108, 2.358)	0.073
	Adjusted	Endovascular treatment	1.890	(0.783, 2.997)	0.001
		Craniotomy	1.278	(−0.290, 2.846)	0.108
12-month MRS	Unadjusted	Endovascular treatment	1.956	(0.886, 3.026)	<0.001***
		Craniotomy	0.913	(−0.442, 2.269)	0.183
	Adjusted	Endovascular treatment	1.982	(0.798, 3.166)	0.001
		Craniotomy	1.491	(−0.186, 3.168)	0.080

Unadjusted model: endovascular compared with conservative treatment and craniotomy compared with conservative treatment. Adjusted model: unadjusted model adjusted for age, sex, history of smoking, history of drinking, diabetes, hypertension.

CI, confidence interval; MRS, modified Rankin scale.

A repeated-measures ANOVA was performed, and the results of post-hoc analysis indicated that the effect of endovascular treatment was better than that of conservative treatment (p < 0.001), and no significant difference was found between the other comparisons. The MRS scores decreased over the three time points (F = 17.481, p < 0.001). No interaction was found between the treatment method and time points (F = 0.297; Supplemental Figure 2).

## Discussion

In this study, we investigated the differences between MIAs and SIAs and evaluated the effects of different treatment modalities on the prognosis of MIA in Chinese patients living on the Tibetan Plateau. The results showed that patients with MIAs were more likely to be women, to smoke, drink alcohol, have hypertension, and have significantly larger aneurysms. Significant differences in treatment results were not found between SIAs and MIAs, indicating that the morbidity type and treatment modality did not affect the disease prognosis at 3, 6, or 12 months. Endovascular treatment showed better performance than did conservative management in patients with MIAs but was not significantly better than craniotomy.

MIAs accounted for 22% of all aneurysms during the study period; a rate of intracranial aneurysms of approximately 7% to 35% has been reported previously.^4,5^ To our knowledge, ours is the first report on the occurrence of MIAs in patients living on the Tibetan Plateau of China. The occurrence of MIAs was associated with the plateau area or related to the living habits or genetic background of Tibetan patients and requires further investigation.

Regarding the risk factors for MIAs, our study findings were generally consistent with those of previous reports. Recent evidence from a meta-analysis indicates that the proportion of female patients, cigarette smoking, age, and primary hypertension are associated with the risk of MIAs.^21^ Our study revealed a significant difference in the percentage of female patients: 75% of patients with MIAs compared with 58.8% of patients with SIA. The mean age of the study population was 52.2 years, which is the period of menopausal transition in most women. The increased risk of MIAs in females may be owing to a decrease in estrogen levels caused by hormonal disorders in postmenopausal or sterilized women.^5,8^

This study showed that smoking and alcohol consumption are risk factors for MIAs. Previous studies have implicated cigarette smoking in MIA development.^8^ Smoking increases oxidative stress, which acts as the initial hemodynamic insult, causing endothelial injury and subsequent inflammation, resulting in aneurysm formation.^22^ A meta-analysis showed that alcohol consumption does not affect MIA formation.^21^ However, in our study, a significant difference was observed in terms of drinking habits (73.5% in MIAs vs. 22.1% in SIAs). This may be owing to the relatively small sample sizes of the included studies. Adequately powered and better-designed studies with long-term follow-up are required to reach firmer conclusions. Hypertension promotes degenerative changes in the vessels during MIA pathogenesis,^23,24^ and drinking can temporarily increase blood pressure levels combined with cerebral vasoconstriction, causing intracranial aneurysm rupture.^21^

There has been some controversy regarding the relationship between age and MIA. With more prolonged exposure to common risk factors for intracranial aneurysms, one study identified an older subpopulation of patients with MIAs than those with SIAs.^16^ In contrast, some scholars have reported that patients with MIAs are relatively younger than those with SIAs, as genetic predispositions (for example, familial intracranial aneurysm and sickle-cell disease) lead to the increased occurrence of MIAs at an earlier age.^25^ In contrast to other studies, we did not find a difference in age between the MIA and SIA subgroups (52.2 vs. 49.8 years, respectively).

Few studies have focused on the specific laboratory features of MIAs. Recently, a study indicated that AB blood group, mixed cell volume, and platelet count were independent risk factors for the occurrence of MIAs.^14^ However, no significant difference was observed between the SIA and MIA subgroups in our study. More powerful and better-designed studies are necessary to reach clearer conclusions.

Overall, our study findings confirm that the risk factors for MIAs in patients living at high altitudes in Tibet are similar to those in other populations. The mechanism underlying the risk factors leading to an increased number of aneurysms reported in this study population living on the Tibetan Plateau requires further investigation.

Previous investigations have demonstrated that aneurysm location, irregular shape, and the presence of bleb formation are strong predictors of a rupture site in MIAs.^10,26^ We found that the aneurysm location with the highest probability of rupture in MIAs was the ICA (36.8%), followed by the MCA (23.5%) and ACA (17.6%), similar to previous reports.^27^ There was no difference with SIA.

In this study, BBAs accounted for 0.5% to 2.0% of ruptured intracranial aneurysms and led to unusually high morbidity and mortality rates.^19^ When aneurysms in the cerebral circulation are excluded, bleeding or rebleeding can be prevented using endovascular or craniotomy treatments.^28^ There are many craniotomy treatments for BBAs, such as direct clipping, clipping after wrapping, or suturing. However, endovascular treatment is preferred because it is convenient and often results in favorable outcomes.^29^ In several studies, endovascular treatment of MIAs has shown good mid- to long-term neurological outcomes.^30^ Various endovascular techniques have been used to treat BBAs, including coil embolization, stenting, stent-assisted coiling, and flow-diverting stents.^31,32^ However, in some studies, the risk of regrowth was found to be higher after endovascular treatment than after craniotomy and clipping.^33^

Despite the increasing number of patients undergoing surgical treatment, 32.4% of our patients chose conservative treatment, which may be associated with high treatment expectations or religious beliefs. Most residents in Tibet are devout followers of Tibetan Buddhism and often choose prayer after the onset of illness, or they seek help at a monastery where lamas judge the need for a hospital visit and even influence decisions regarding surgery.^34^ Patients who choose conservative treatment may do so for reasons unrelated to their own health considerations. The lifestyle customs and religious culture of Tibetan patients should be considered, their cultural beliefs should be respected, and education and guidance should be conducted in a stepwise manner based on education level to ensure that patients and their families fully understand the prevention and treatment of brain aneurysm to improve the cure rate of affected patients. In this study, endovascular treatment in patients with MIAs was superior to conservative management. However, no significant difference was observed compared with craniotomy. Therefore, craniotomy is the second modality of choice in this population. Further studies with larger numbers of patients are necessary to establish better criteria for determining which patients would benefit from craniotomy or endovascular treatment.

### Limitations

In this study, we performed a comparative effectiveness analysis based on real-world data to investigate the treatment modality that would most benefit patients with intracranial aneurysms. However, this study has some limitations. First, the study was confined to only one center on the Tibetan Plateau, and a certain degree of bias may exist in the results. Second, the incidence and rupture rates of MIAs could not be accurately ascertained because a considerable proportion of patients with SAH may have died before reaching the hospital, and the actual number is difficult to estimate in Tibet. Patients predicted to have unfavorable outcomes were treated at local hospitals and were not transferred. Only patients with mild symptoms were treated at our center. Additionally, further analysis of patients’ other clinical data at follow-up was not performed. Moreover, it would be valuable in future studies to select patients with multiple aneurysms from low-altitude regions for comparisons with those in Tibet. Finally, the treatment modalities were based on the patient’s preference rather than on a randomized controlled trial; thus, the disease outcomes varied depending on the treatment modality.

## Conclusion

Overall, our study results showed that smoking, alcohol consumption, and hypertension may be risk factors for MIAs, and endovascular treatment was better than conservative management for treating MIAs but not significantly different from craniotomy. However, this was a single-center study and lacked controls from the plateau region, and the choice of treatment was based on the patients’ preferences. Therefore, the mechanisms via which risk factors lead to an increase in the number of aneurysms reported in our study population living on the Tibetan Plateau should be comprehensively investigated and compared in the future. Further studies with larger cohorts and more detailed demographic surveys are required to establish criteria to determine which patients are more suitable for craniotomy or endovascular treatment.

## Supplemental Material

sj-pdf-1-imr-10.1177_03000605241306870 - Supplemental material for Clinical characteristics and prognosis of patients with multiple intracranial aneurysms living on the Tibetan Plateau of China Supplemental material, sj-pdf-1-imr-10.1177_03000605241306870 for Clinical characteristics and prognosis of patients with multiple intracranial aneurysms living on the Tibetan Plateau of China by Dongliang Wang, Jiuqi Wuli, Xudong Cao, Bin Wang, Zeng Ren, Yu Weng and Kexue Wu in Journal of International Medical Research

## Data Availability

The datasets generated during and/or analyzed during the current study are available from the corresponding author upon reasonable request.

## References

[1] Claassen J Park S. Spontaneous subarachnoid haemorrhage. Lancet 2022; 400: 846–862. 35985353 10.1016/S0140-6736(22)00938-2 PMC9987649

[2] Kim JH Lee KY Ha SW , et al. Prevalence of Unruptured Intracranial Aneurysms: A Single Center Experience Using 3T Brain MR Angiography. Neurointervention 2021; 16: 117–121. 33906286 10.5469/neuroint.2021.00024 PMC8261115

[3] Ferre L Filippi M Esposito F. Involvement of Genetic Factors in Multiple Sclerosis. Front Cell Neurosci 2020; 14: 612953. 33335478 10.3389/fncel.2020.612953 PMC7735985

[4] Liberato ACP Xu J Montes D , et al. Multivariable analysis on factors associated with aneurysm rupture in patients with multiple intracranial aneurysms. Emerg Radiol 2020; 27: 487–494. 32458143 10.1007/s10140-020-01790-5

[5] McDowell MM Zhao Y Kellner CP , et al. Demographic and clinical predictors of multiple intracranial aneurysms in patients with subarachnoid hemorrhage. J Neurosurg 2018; 128: 961–968. 28598275 10.3171/2017.1.JNS162785

[6] Sai Kiran NA Raj V Sivaraju L , et al. Outcome of Microsurgical Clipping for Multiple Versus Single Intracranial Aneurysms: A Single-Institution Retrospective Comparative Cohort Study. World Neurosurg 2020; 143: e590–e603. 32781147 10.1016/j.wneu.2020.08.019

[7] Fung C Mavrakis E Filis A , et al. Anatomical evaluation of intracranial aneurysm rupture risk in patients with multiple aneurysms. Neurosurg Rev 2019; 42: 539–547. 29959638 10.1007/s10143-018-0998-1

[8] Jabbarli R Dinger TF Darkwah Oppong M , et al. Risk Factors for and Clinical Consequences of Multiple Intracranial Aneurysms: A Systematic Review and Meta-Analysis. Stroke 2018; 49: 848–855. 29511128 10.1161/STROKEAHA.117.020342

[9] Jeon HJ Lee JW Kim SY , et al. Morphological parameters related to ruptured aneurysm in the patient with multiple cerebral aneurysms (clinical investigation). Neurol Res 2014; 36: 1056–1062. 24852695 10.1179/1743132814Y.0000000393

[10] Jiang H Weng YX Zhu Y , et al. Patient and aneurysm characteristics associated with rupture risk of multiple intracranial aneurysms in the anterior circulation system. Acta Neurochir (Wien) 2016; 158: 1367–1375. 27165300 10.1007/s00701-016-2826-0

[11] Wang X Wang C Zhu T , et al. Persistent organic pollutants in the polar regions and the Tibetan Plateau: A review of current knowledge and future prospects. Environ Pollut 2019; 248: 191–208. 30784838 10.1016/j.envpol.2019.01.093

[12] Xing CY Serrador JM Knox A , et al, Cerebral Blood Flow. Oxygen Delivery, and Pulsatility Responses to Oxygen Inhalation at High Altitude: Highlanders vs. Lowlanders. Front Physiol 2019; 10: 61. 30792663 10.3389/fphys.2019.00061 PMC6375252

[13] Dang SN Wang ZJ Kang YJ , et al. Study on the dietary pattern assessed with semi-quantitative food frequency questionnaire among rural Tibetan women with children younger than 2 years in Lhasa city. Zhonghua Liu Xing Bing Xue Za Zhi 2010; 31: 394–399. 20513282

[14] Dinger TF Darkwah Oppong M Park C , et al. Development of multiple intracranial aneurysms: beyond the common risk factors. J Neurosurg 2022; 137: 1056–1063. 35120308 10.3171/2021.11.JNS212325

[15] Xin WQ Sun PJ Li F , et al. Risk factors involved in the formation of multiple intracranial aneurysms. Clin Neurol Neurosurg 2020; 198: 106172. 32942133 10.1016/j.clineuro.2020.106172

[16] Juvela S. Risk factors for multiple intracranial aneurysms. Stroke 2000; 31: 392–397. 10657411 10.1161/01.str.31.2.392

[17] Tejus MN Singh D Jagetia A , et al. Endovascular Nuances in Management of Multiple Intracranial Aneurysms. Neurol India 2019; 67: 1062–1065. 31512636 10.4103/0028-3886.266249

[18] Lv X Li Y Liu A , et al. Endovascular management of multiple cerebral aneurysms in acute subarachnoid hemorrhage associated with fenestrated basilar artery. A case report and literature review. Neuroradiol J 2008; 21: 137–142. 24256763 10.1177/197140090802100120

[19] Hao X Li G Ren J , et al. Endovascular Patch Embolization for Blood Blister-Like Aneurysms in Dorsal Segment of Internal Carotid Artery. World Neurosurg 2018; 113: 26–32. 29325955 10.1016/j.wneu.2018.01.014

[20] Von Elm E Altman DG Egger M ; STROBE Initiative et al. The Strengthening the Reporting of Observational Studies in Epidemiology (STROBE) statement: guidelines for reporting observational studies. Ann Intern Med 2007; 147: 573–577. 17938396 10.7326/0003-4819-147-8-200710160-00010

[21] Behrooz M Vaghef-Mehrabany E Ostadrahimi A. Different spexin level in obese vs. normal weight children and its relationship with obesity related risk factors. Nutr Metab Cardiovasc Dis 2020; 30: 674–682. 32139252 10.1016/j.numecd.2019.11.008

[22] Ho AL Lin N Frerichs KU , et al. Smoking and Intracranial Aneurysm Morphology. Neurosurgery 2015; 77: 59–66; discussion 66. 25839377 10.1227/NEU.0000000000000735

[23] Tada Y Wada K Shimada K , et al. Roles of hypertension in the rupture of intracranial aneurysms. Stroke 2014; 45: 579–586. 24370755 10.1161/STROKEAHA.113.003072 PMC3935821

[24] McCormick WF Schmalstieg EJ. The relationship of arterial hypertension to intracranial aneurysms. Arch Neurol 1977; 34: 285–287. 856126 10.1001/archneur.1977.00500170039006

[25] Jabbarli R Dinger TF Pierscianek D , et al. Intracranial aneurysms in sickle cell disease: a systematic review and case-control study. Curr Neurovasc Res 2019; 16: 11. 10.2174/1567202616666190131160847 30706782

[26] Greving JP Wermer MJH Brown RD , et al. Development of the PHASES score for prediction of risk of rupture of intracranial aneurysms: a pooled analysis of six prospective cohort studies. Lancet Neurol 2014; 13: 59–66. 24290159 10.1016/S1474-4422(13)70263-1

[27] Murayama Y Takao H Ishibashi T , et al. Risk Analysis of Unruptured Intracranial Aneurysms. Stroke 2016; 47: 365–371. 26742803 10.1161/STROKEAHA.115.010698

[28] Diaz O Rangel-Castilla L. Endovascular treatment of intracranial aneurysms. Handb Clin Neurol 2016; 136: 1303–1309. 27430470 10.1016/B978-0-444-53486-6.00067-3

[29] Ji T Guo Y Huang X , et al. Current status of the treatment of blood blister-like aneurysms of the supraclinoid internal carotid artery: A review. Int J Med Sci 2017; 14: 390–402. 28553172 10.7150/ijms.17979 PMC5436482

[30] Panigrahi M Patel C Koradia P , et al. Contralateral Clipping of Multiple Intracranial Aneurysms. Adv Tech Stand Neurosurg 2022; 44: 161–173. 35107678 10.1007/978-3-030-87649-4_8

[31] Andic C Aydemir F Kardes O , et al. Single-stage endovascular treatment of multiple intracranial aneurysms with combined endovascular techniques: is it safe to treat all at once? J Neurointerv Surg 2017; 9: 1069–1074. 27977003 10.1136/neurintsurg-2016-012745

[32] Chen J Tong X Feng X , et al. Management of Unruptured Small Multiple Intracranial Aneurysms in China: A Comparative Effectiveness Analysis Based on Real-World Data. Front Neurol 2021; 12: 736127. 35153970 10.3389/fneur.2021.736127 PMC8830354

[33] Okada T Ishikawa T Moroi J , et al. Timing of retreatment for patients with previously coiled or clipped intracranial aneurysms: Analysis of 156 patients with multiple treatments. Surg Neurol Int 2016; 7: S40–S48. 26862460 10.4103/2152-7806.173570 PMC4722515

[34] Dai Z Liu J Chen L. Clinical characteristics and application value of risk prediction models of acute appendicitis in rural Tibet: a retrospective study. Rural Remote Health 2023; 23: 7709. 37856895 10.22605/RRH7709

