# Supplementary material for: Clinical characteristics and prognosis of patients with multiple intracranial aneurysms living on the Tibetan Plateau of China
Source: J Int Med Res. 2024 Dec 29;52(12):03000605241306870. doi: 10.1177/03000605241306870 (PMC11686787; doi:10.1177/03000605241306870)
Supplement: sj-pdf-1-imr-10.1177_03000605241306870 - Supplemental material for Clinical characteristics and prognosis of patients with multiple intracranial aneurysms living on the Tibetan Plateau of China [file sj-pdf-1-imr-10.1177_03000605241306870.pdf]

Supplemental Table 1. Specific characteristics of aneurysms in MIA patients

Variables	Total (n = 157)	Number of aneurysms 2 (n = 105)	3 (n = 39)	≥ 4 (n = 13)
Size, mm	6.3 ± 1.8	6.5 ± 1.8	5.8 ± 1.8	5.3 ± 1.6
Location				
ACA	30 (19.1%)	22 (73.3%)	6 (20%)	2 (6.7%)
ICA	56 (35.7%)	35 (62.5%)	17 (30.4%)	4 (7.1%)
MCA	62 (39.5%)	44 (71%)	13 (21%)	5 (8.1%)
VBA	9 (5.7%)	4 (44.4%)	3 (33.3%)	2 (22.2%)

MIA, multiple intracranial aneurysm; ACA, anterior cerebral artery; ICA, internal carotid artery; MCA, middle cerebral artery; VBA, vertebral artery

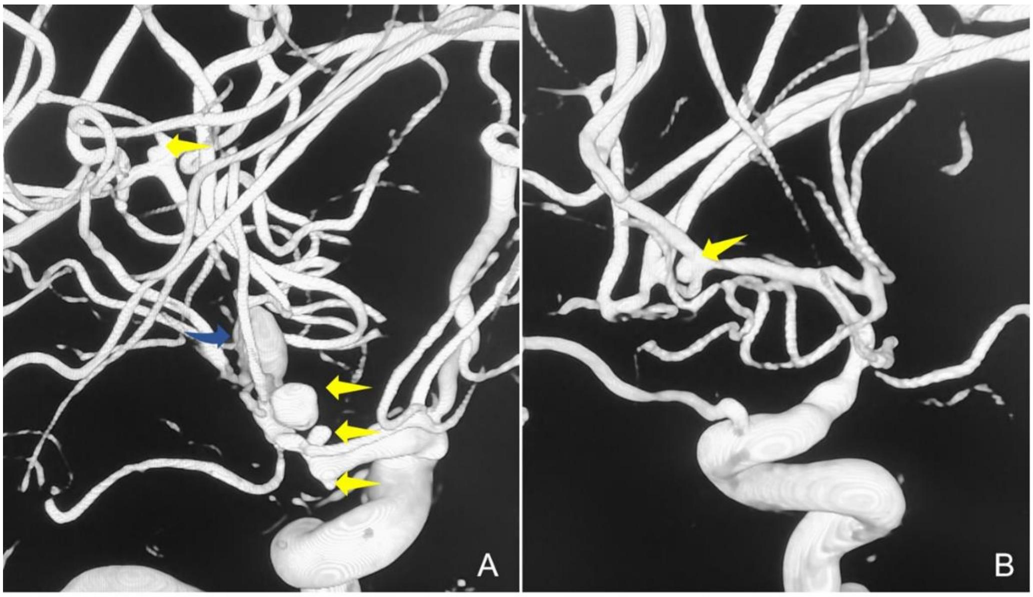

Supplemental Figure 1. Multiple intracranial aneurysms (MIAs) of a representative case. (A) Left internal carotid artery (ICA) angiography of the patient with yellow arrows indicating multiple cystic aneurysms and blue arrows indicating fusiform aneurysms of the middle cerebral artery (MCA). (B) Right ICA angiography of the same patient with arrows indicating aneurysms.

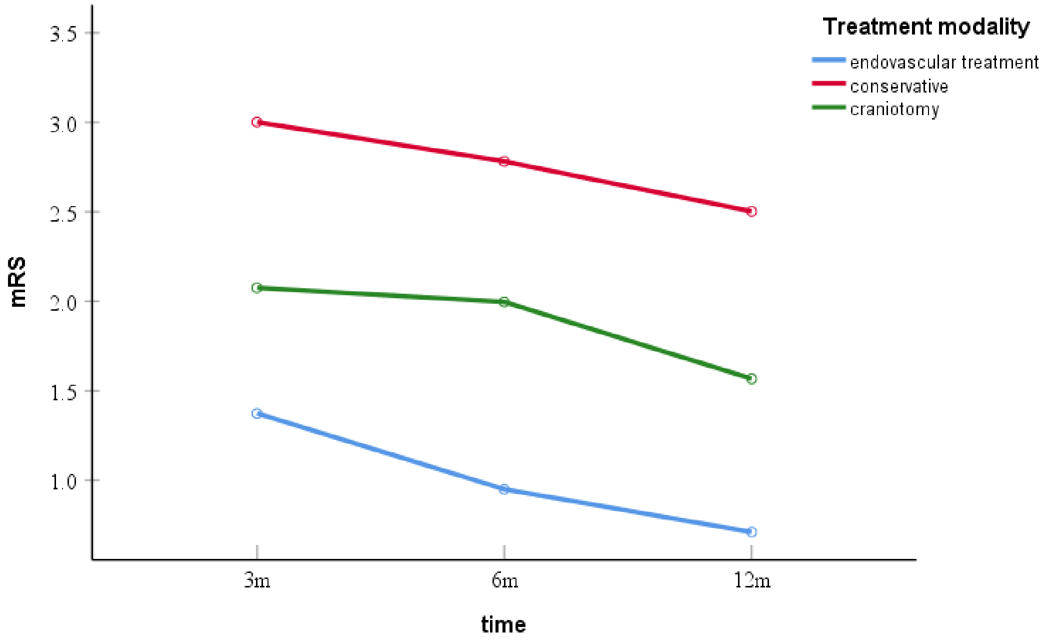

Supplemental Figure 2. Effects of treatment modality on Modified Rankin Scale (MsRS) score in multiple intracranial aneurysm (MIA) patients
